# In Situ Thai *Apis mellifera* Propolis Film as Potential Protective Phytopharmaceuticals Against UVB-Induced HaCaT Keratinocyte Damage

**DOI:** 10.3390/ph19050680

**Published:** 2026-04-27

**Authors:** Takron Chantadee, Anyamanee Chatsirisupachai, Ampai Phrutivorapongkul, Sunee Chansakaow, Sasithorn Sirilun, Onusa Thamsermsang

**Affiliations:** 1 Department of Pharmaceutical Sciences, Faculty of Pharmacy, Chiang Mai University, Chiang Mai 50200, Thailand; takron.chantadee@cmu.ac.th (T.C.); ampai.phrutiv@cmu.ac.th (A.P.); sunee.c@cmu.ac.th (S.C.); sasithorn.s@cmu.ac.th (S.S.); 2 Center of Excellence in Pharmaceutical Nanotechnology, Chiang Mai University, Chiang Mai 50200, Thailand; 3 Princess Srisavangavadhana Faculty of Medicine, Chulabhorn Royal Academy, Bangkok 10210, Thailand; anyamanee.cha@cra.ac.th

**Keywords:** propolis, bioactive compounds, in situ film-forming systems, UVB radiation, wound healing, human keratinocytes, cutaneous applications

## Abstract

**Background/Objectives**: Propolis is well recognized for its antioxidant, anti-inflammatory, and wound-healing properties, supporting its cutaneous application in phytopharmaceuticals for the management of ultraviolet B (UVB)-induced skin damage. However, the application of propolis is limited by its intense coloration, stickiness, and poor user convenience. In situ film-forming systems (FFS) represent a novel dosage form designed to overcome these challenges, although efficacy data for using FFS remains limited. Consequently, this study aimed to develop a propolis-based FFS and evaluate its efficacy in mitigating UVB-irradiated HaCaT keratinocytes. **Methods**: *Apis mellifera* propolis was macerated and analyzed for total phenolic content (TPC) and total flavonoid content (TFC), radical scavenging activity (DPPH assay), and nitric oxide scavenging capability. Bioactive compounds were identified using high-performance liquid chromatography analysis (HPLC). The propolis extract was formulated into FFS and investigated on UVB-damaged HaCaT keratinocytes. An MTT viability assay, propidium iodide flow cytometry for cell cycle analysis, and a scratch wound healing assay were used to evaluate the therapeutic effects of the FFS. **Results**: The 72 h macerated propolis extract contained high levels of TPC, TFC, and targeted phytochemicals. The propolis extract exhibited free radical scavenging and nitric oxide inhibitory activities. Seven formulations exhibited suitable performance, with formulation F7 (FFS-F7) demonstrating superior drying time and dose-dependent free radical scavenging. Notably, FFS-F7 (≥12.5 µg/mL) significantly enhanced HaCaT proliferation, mitigated UVB-induced cell cycle arrest, reduced cellular damage, and accelerated wound closure. **Conclusions**: This study successfully developed an FFS that not only overcomes these physical drawbacks but also preserves the biological activity of the extract. The significant protective and restorative effects against UVB-induced HaCaT damage demonstrate the therapeutic potential of Thai *Apis mellifera* propolis and establish the FFS as a versatile base with the potential for delivering other bioactive compounds.

## 1. Introduction

The global incidence of skin diseases associated with ultraviolet radiation (UVR) exposure has escalated significantly, posing a substantial burden on healthcare systems. UVR, a component of sunlight, is a ubiquitous environmental factor classified as carcinogenic to humans by the International Agency for Research on Cancer (IARC) [1] and includes UVA (315–400 nm) and UVB (280–315 nm) [2]. Among these, UVB radiation is of particular concern due to its predominant absorption within the epidermis, where it directly induces cellular DNA damage and promotes oxidative stress through the generation of reactive oxygen species (ROS) [3]. UVB-induced oxidative and genotoxic stress disrupts cell cycle progression, thereby impairing wound repair processes and leading to cellular dysfunction. Consequently, these cascades contribute to acute and chronic dermatitis, characterized by inflammation and barrier disruption, as well as photoaging and the development of skin cancers [3,4,5]. Despite an extensive understanding of these mechanisms, translating this knowledge into effective strategies to mitigate UVB-induced cellular dysfunction remains to be elucidated.

Human keratinocytes are the predominant epidermal cell type and are responsible for maintaining barrier integrity and coordinating tissue repair responses to environmental insults, including UVB irradiation. UV-induced DNA damage activates cellular surveillance mechanisms that detect genomic lesions and initiate DNA repair through cell-cycle checkpoints at G1 and G2 phases [6]. However, excessive or persistent damage can overwhelm these protective responses, leading to cell cycle arrest and impaired cellular function [7]. In addition, oxidative stress disrupts cellular redox homeostasis, including NAD(P)H-dependent metabolic processes, thereby compromising cell viability and regenerative capacity [8]. These cellular dysfunctions collectively impair wound healing processes and contribute to the development of chronic skin damage [9]. Given the multifaceted damage caused by UVB-induced oxidative stress and genotoxicity, natural bioactive compounds have increasingly been investigated for their photoprotective and skin-repair properties. However, their effective delivery and sustained activity in the skin remain challenging.

Propolis, a resinous material produced by honeybees, has been widely utilized as a traditional, complementary, and integrative medicine (TCIM) for the management of various skin conditions, including infections and eczema, and for promoting wound healing. Its biological activities are driven by phenolic acids, flavonoids, and other bioactive compounds, which exhibit antioxidant, anti-inflammatory, and antimicrobial effects [10]. However, the chemical composition of propolis varies considerably depending on botanical origin and environmental factors, resulting in diverse profiles of bioactive compounds, including phenolic acids (e.g., gallic acid, caffeic acid, p-coumaric acid, and ferulic acid) and flavonoids (e.g., luteolin, quercetin, apigenin, caffeic acid phenethyl ester, and chrysin) [11,12,13]. These compounds are commonly used as representative chemical markers for the characterization and standardization of propolis [14,15]. Despite these promising biological properties, the therapeutic application of propolis is often limited by its inherent physicochemical characteristics, including poor solubility, instability, and inadequate retention at the site of application, necessitating the development of advanced delivery systems to enhance its clinical utility.

The in situ film-forming system (FFS) represents a novel pharmaceutical platform that overcomes the limitations of traditional topical dosage forms while improving patient compliance. Conventional solutions offer ease of application due to their low viscosity and non-greasy nature but suffer from poor skin residence time. In contrast, semi-solid formulations such as creams and ointments provide prolonged skin retention but are frequently associated with undesirable stickiness, limited spreadability, and staining of clothing, particularly when incorporating highly pigmented active ingredients such as propolis. To overcome these challenges, FFS is designed to undergo a liquid-to-film transformation upon skin contact. The formulation is applied as a low-viscosity liquid via spray or roller and subsequently forms a thin, flexible film on the skin as volatile solvents evaporate, providing a protective barrier against environmental contaminants and mechanical friction. FFS typically consists of film-forming polymers, plasticizers, volatile solvents, and other formulation components [16]. Because of its solution-based formulation, FFS provides greater ease of use than traditional dosage forms such as gels or emulsions while enabling rapid drying and a non-sticky layer after application [17,18].

Several commercial products have utilized FFS technology for wound management. Examples include Liquiplast, a solution-based formulation using pyroxylin as the film-forming agent [19], and Hansaplast spray plaster, which employs acrylic copolymer and polyurethane [20]. In active-loaded systems, Axiron^®^ incorporates testosterone as the active pharmaceutical ingredient (API) with polyvinylpyrrolidone K90 as the polymer base [21]. Furthermore, a previous study has investigated various FFS applications, such as chlorhexidine gluconate spray for topical disinfection. This formulation, utilizing Eudragit^®^ S100, Eudragit^®^ L100, and polyvinyl alcohol, demonstrated the formulation of a transparent thin film with rapid drying characteristics [22].

Driven by these advancements, this study aims to develop a spray FFS using appropriate polymers and excipients to achieve optimal film performance. To ensure durability and transparency, both water-soluble and water-insoluble polymers, including Eudragit RS, Polyvinyl Alcohol, and Polyvinylpyrrolidone, were evaluated [16]. To achieve water-resistant properties, water-insoluble polymers were prioritized; however, as these often result in brittle films, plasticizers such as Propylene glycol, Dibutyl phthalate [16], and N-methyl-2-pyrrolidone [23] were incorporated to enhance flexibility. Volatile and non-irritating solvents, including ethanol, isopropyl alcohol, and butanol, were considered for rapid film formation [16]. Additionally, antimicrobial agents such as sulfur dioxide, chlorhexidine, and povidone-iodine may be included [24]. Notably, certain agents like n-butyl cyanoacrylate serve a dual role as both an antimicrobial and an adhesive [25]. Consequently, the final formulation selected for this study consists of Eudragit RS, n-butyl cyanoacrylate, ethanol, and N-methyl-2-pyrrolidone (NMP). Interestingly, the huge challenge is that the efficacy data of using an in situ forming system remains limited. Most of the previous studies aimed to develop suitable physicochemical properties and their behaviors. Less data on the efficacy and safety of using in situ-forming systems is available. Thus, this study aims not only to develop an effective delivery system but also to evaluate the therapeutic performance of propolis as a model drug.

Another reason that renders FFS interesting in this case is the limitation of using propolis for UV-induced skin damage. It is difficult to formulate propolis preparations for cutaneous application on compromised and hypersensitive skin. This study explored the advantages of FFS to overcome drawbacks and limitations associated with traditional topical formulations, such as creams and ointments. FFS transitions from a liquid or semi-liquid state to a solid or gel-like state in response to changes in physiological conditions. This enables FFS to provide uniform, sustained, and accurate drug delivery while enhancing bioavailability, reducing irritation, and accelerating wound healing [20,26]. Furthermore, FFS can be applied to irregular skin surfaces and areas of varying sizes while providing a protective barrier on the treated site, shielding it from friction, moisture, contaminants, and contact with clothing.

In this study, phytopharmaceuticals refer to natural product-derived bioactive compounds, particularly propolis-derived constituents incorporated into a formulation system with demonstrated biological activity. Therefore, this study aims to develop a topical propolis-loaded FFS derived from *Apis mellifera* propolis sourced in northern Thailand and to investigate its protective effects and wound-healing potential in HaCaT keratinocytes.

## 2. Results

### 2.1. Extraction Yield, Total Phenolic Content, and Total Flavonoid Content of the Propolis Extract

The 72 h macerated propolis extract had a yield of 51.19% (*w*/*w*). The total phenolic content was approximately 18.70 GAE mg/g DWE, and the total flavonoid content was 3.01 QE mg/g DWE, indicating the substantial presence of phenolic and flavonoid compounds in the extract (Table 1).

### 2.2. Targeted Phenolic and Flavonoid Compounds in Thai Apis mellifera Propolis Extract

**Figure 1:**
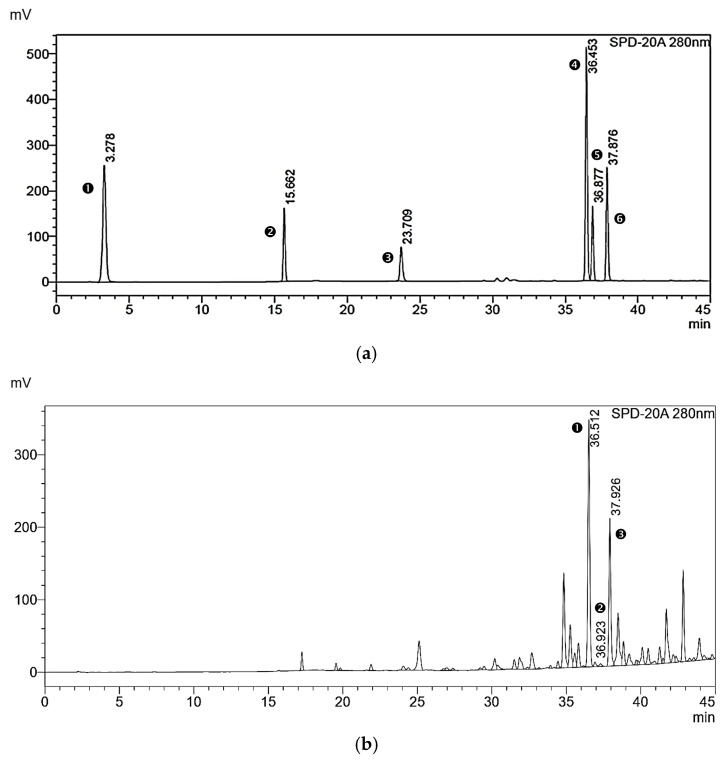
The HPLC chromatograms of standard compound mixture and propolis extract (n = 5): (**a**) Chromatographic fingerprint of six reference compounds. Peak details: 1: Gallic acid (RT: 3.278 min); 2: p-coumaric acid (RT: 15.662 min); 3: Quercetin (RT: 23.709 min); 4: Chrysin (RT: 36.453 min); 5: Caffeic acid phenethyl ester (RT: 36.877 min); 6: Galangin (RT: 37.876 min). (**b**) The overall HPLC chromatograms of propolis extracts from the 72 h maceration method. Peak details: 1: Chrysin (RT: 36.512 min); 2: Caffeic acid phenethyl ester (RT: 36.923 min); and 3: Galangin (RT: 37.926 min).

HPLC analysis revealed noticeable separation of six phenolic and flavonoid compounds in the standard mixture (Figure 1a). Chrysin, caffeic acid phenethyl ester, and galangin were established as the predominant constituents in the propolis extract. However, gallic acid, p-coumaric acid, and quercetin (Figure 1b) were not found in the propolis sample. Quantitative analysis of the extract obtained after 72 h of maceration revealed the presence of chrysin (CS) (43.72 µg/mL), galangin (GG) (40.39 µg/mL), and caffeic acid phenethyl ester (CAPE) (1.34 µg/mL) (Table 2).

### 2.3. Antioxidant and Nitric Oxide Scavenging Activities of Propolis Extract

**Figure 2:**
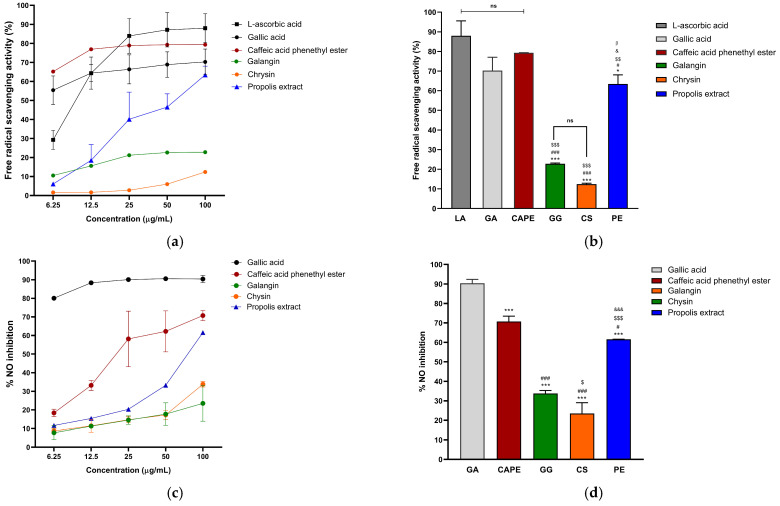
The antioxidant and nitric oxide scavenging activities of 72 h macerated propolis extract. All data were represented as mean ± SD with triplicate experiments. (**a**) Dose-dependent free radical scavenging activities of propolis extracts and reference markers. (**b**) Comparison of the highest doses of propolis extracts with positive control groups and reference markers using ANOVA (Bonferroni post hoc test) (* *p* < 0.05, *** *p* < 0.001 vs. L-ascorbic acid; ^#^ *p* < 0.05, ^###^ *p* < 0.01 vs. gallic acid; ^$$$^ *p* < 0.001, ^$$^ *p* < 0.01 vs. caffeic acid phenethyl ester; ^&^ *p* < 0.05 vs. galangin; ^β^ *p* < 0.05 vs. chrysin; ns: not significant). (**c**) Nitric oxide scavenging activity of the propolis extract at different concentrations. (**d**) Analysis of variance (ANOVA) comparison of the highest doses of propolis extracts and reference markers, followed by Bonferroni post hoc test (*** *p* < 0.001 vs. L-ascorbic acid; ^#^ *p* < 0.05, ^###^ *p* < 0.001 vs. caffeic acid phenethyl ester; ^$^ *p* < 0.05, ^$$$^ *p* < 0.001 vs. galangin; ^&&&^ *p* < 0.001 vs. chrysin).

As shown in Figure 2a, the propolis extract demonstrated superior and dose-dependent free radical scavenging with superior antioxidant potential compared to chrysin and galangin (25–100 µg/mL). No significant differences were observed among the positive controls (L-ascorbic acid and gallic acid) and caffeic acid phenethyl ester. The antioxidant potential of the propolis extract at 100 μg/mL was significantly higher than galangin and chrysin (*p* < 0.05) and comparable to positive control groups and caffeic acid phenethyl ester (Figure 2b and Table 1).

As a preliminary assessment, the nitric oxide (NO) scavenging activity of the propolis extract was evaluated. The results showed increasing activity with concentration (Figure 2c). At the highest concentration (100 μg/mL), gallic acid exhibited the strongest NO scavenging activity among all tested compounds, followed by caffeic acid phenethyl ester and the propolis extract, while galangin and chrysin were comparatively less effective (Figure 2d; Table 1).

### 2.4. Development of FFS

#### 2.4.1. FFS Formulation and Preparation

A propolis-loaded FFS was developed using a full factorial design of experiments (DoE) approach to identify an optimized formulation for topical delivery. Fourteen formulations were generated, varying the concentrations of Eudragit^®^ RS 100 and cosolvent composition. Among these, seven formulations (F1–F7), which did not contain n-butyl cyanoacrylate, exhibited complete dissolution and remained physically stable over 24 h. In contrast, formulations containing n-butyl cyanoacrylate (F8–F14) showed instability, primarily due to polymer aggregation and precipitation. Seven formulations (FFS-F1–F7) consisted of Eudragit^®^ RS 100, N-methyl-2-pyrrolidone (NMP), dimethyl sulfoxide (DMSO), and absolute ethanol, with a fixed propolis content of 0.5% (*w*/*w*). The absence of n-butyl cyanoacrylate contributed to improved formulation homogeneity, likely due to reduced susceptibility to premature polymerization and phase separation in the presence of trace moisture (see Section 4.8.1). These findings indicate that solvent composition and polymer compatibility play critical roles in determining formulation stability and support the selection of F1–F7 for further evaluation of film-forming performance.

#### 2.4.2. Evaluation of FFS Solution: Apparent Stability (24 H Assessment), pH, and Viscosity

**Figure 3:**
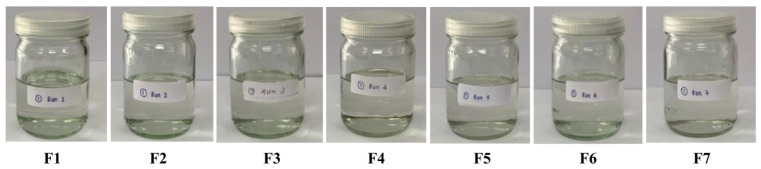
The visual appearances of the homogeneous in situ propolis-loaded film-forming solutions (**F1**–**F7**).

According to the physical appearance evaluation, formulations F1–F7, which did not contain n-butyl cyanoacrylate, exhibited clear, homogeneous solutions without precipitation or phase separation (Figure 3). In contrast, formulations F8–F14 were slightly turbid and predominantly contained precipitates that dispersed uniformly upon shaking. This rendered F8–F14 unsuitable for development as an in situ film-forming spray formulation. Regarding the pH evaluation (Table 3), all formulations exhibited a pH in the range of 5.5–7.

#### 2.4.3. Film-Forming Behavior (Drying Time) and Post-Drying Stickiness Evaluation

All films exhibited smooth, flexible films with non-sticky surfaces. Formulations FFS-F1–F7 formed clear films, whereas F8–F14 resulted in translucent films (Table 3), indicating differences in film uniformity and structural homogeneity. The drying time of F1–F7 ranged from 3.01 to 5.39 min, whereas significantly longer drying times (5.75–41.27 min) were observed for F8–F14 containing n-butyl cyanoacrylate.

**Figure 4:**
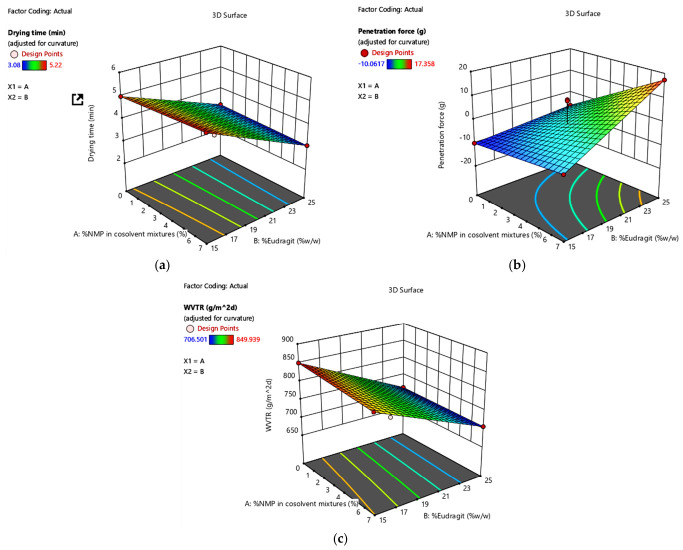
A 3D response surface plots of film performance parameters as functions of %N-methyl-2-pyrrolidone (NMP) in the cosolvent mixture and Eudragit^®^ RS 100 concentration (%*w*/*w*). (**a**) Drying time. (**b**) Penetration force. (**c**) Water vapor transmission rate (WVTR).

It was found that formulation F7 used the lowest drying time at 3.08 min, which was composed of the highest concentration of Eudragit^®^ RS 100. While formulation F4, which has the lowest concentration of Eudragit^®^ RS 100, took the longest drying time at 5.22 min (Table 3). Since the without n-butylcyanoacrylate series provides a more suitable solution-state stability over 24 h, a clear forming film, and faster film-forming behavior, F1–F7 were selected to analyze in advance (Figure 4).

#### 2.4.4. Mechanical Properties Evaluation

It was found that the amount of Eudragit^®^ RS 100 and the proportion of solvent in the formulation have an effect on the mechanical properties of the film (Figure 4b). Formulations F2, F5, and F6, which are formulations with the same solvent proportion and amount of Eudragit^®^ RS 100, had similar penetration force at 8.648, 8.904, and 8.398 g, respectively. While F1, F3, and F4 had negative penetration force, meaning the penetration force of the film on agar gel was lower than that of the single agar gel.

#### 2.4.5. Water Vapor Transmission Rate (WVTR)

All formulations F1–F7 had lower WVTR than the control group (706.5006–849.939 and 1091.764 g/m^2^d, respectively). It can be stated that the formed film can retain water vapor on the skin to a certain level when compared to without the film. Formulation F7 had the lowest WVTR, which is 706.5006 g/m^2^d, and formulation F1 had the highest value, which is 849.939 g/m^2^d.

### 2.5. Antioxidant Activity of Propolis-Loaded FFS and Antioxidant Release Profile (Antioxidant Activity over Time)

**Figure 5:**
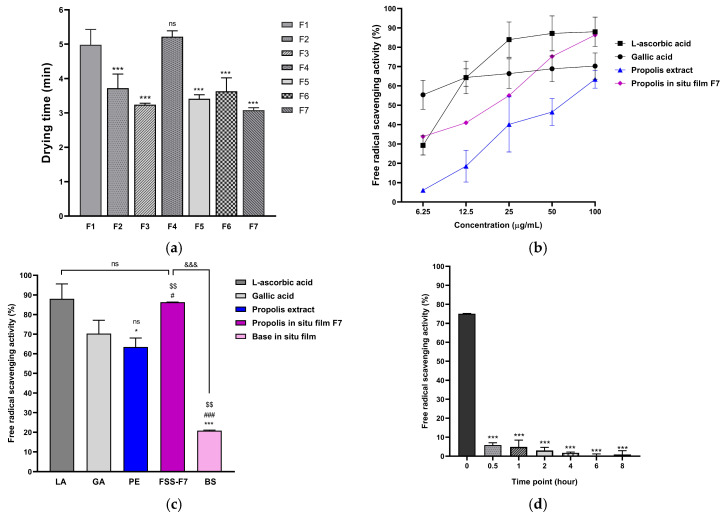
Drying time, antioxidant activities, and release profile of propolis-loaded FFS. (**a**) Drying time of propolis-loaded films from seven in situ formulations. Data represent the mean ± SD of triplicate experiments analyzed using ANOVA followed by Dunnett’s post hoc test (*** *p* < 0.001 vs. F1; ns: not significant). (**b**) Dose-dependent antioxidant activity of the propolis-in situ solution. (**c**) Antioxidant activity at the highest concentration of propolis extract, in situ F7 formulation (FFS-F7), and base in situ compared with L-ascorbic acid and gallic acid. Statistical analysis was performed using one-way ANOVA followed by Bonferroni’s post hoc test (* *p* < 0.05, *** *p* < 0.001 vs. L-ascorbic acid; ^#^ *p* < 0.05, ^###^ *p* < 0.001 vs. gallic acid; ^$$^ *p* < 0.01 vs. propolis extract; ^&&&^ *p* < 0.001 vs. base in situ; ns: not significant). (**d**) Antioxidant release profile (antioxidation activity over time) of the propolis-loaded film evaluated at different time intervals using Dunnett’s post hoc test (*** *p* < 0.05 vs. time 0 h).

Based on the findings for physical characteristics and drying time, the developed formulations not only showed homogeneity and stability in the solution state but also showed a preferred in situ forming system performance and film characteristics such as pH, drying time, non-stickiness film, highest mechanical strength, and suitable WVTR of F7. These properties improve the pain points of using propolis in other dosage forms since its intense coloration and stickiness, leading to ease of staining on garments and various objects. The outstanding feature, which indicates the ease of use (represented by drying time as shown in Figure 5a) and minimizes the mentioned pain points, is FFS-F7. Therefore, FFS-F7 was selected to deeply investigate the therapeutic performance involving antioxidant potential and drug release determination. FFS-F7 exhibited a dose-dependent response and demonstrated superior efficacy compared to the propolis extract alone. FFS-F7 at 50 and 100 µg/mL had higher free radical scavenging potential than gallic acid but was less effective than L-ascorbic acid (Figure 5b). No statistically significant difference between the propolis extract and gallic acid (Figure 5c). FFS-F7 at 100 µg/mL provided an antioxidant effect comparable to that of L-ascorbic acid and higher than that of gallic acid.

Further investigation of the time-dependent antioxidant activity of FFS-F7 was assessed to provide the antioxidant release profile, which can ensure that the loaded propolis extract can diffuse out from the formed film and exhibit antioxidant activity (Figure 5d). This method can provide a more comprehensive assessment of the therapeutic performance than using the traditional drug release method, since propolis has various active components. Within the first 0–0.5 h, FFS-F7 demonstrated a clear initial burst of free radical scavenging activity, releasing approximately 75% inhibition at the initial time point (0 h). This scavenging activity was rapidly consumed, decreasing to about 5% by 0.5 h, which reflects the intended rapid-release profile for acute antioxidant protection. These confirm that FFS-F7 is an immediate-release dosage form that is able to provide antioxidation activity at the time of use, together with the mentioned advantages. Accordingly, the formulation exhibited a rapid antioxidant release profile immediately following film formation. Consistent with the Quality Target Product Profile (QTPP), the system demonstrated immediate-release behavior characterized by an initial burst release of antioxidant activity followed by a rapid decline. Moreover, the film-forming characteristics enabled strong skin adhesion without visible residue while allowing rapid diffusion of antioxidant compounds from the formed film. Thus, the formulation was designed to provide rapid antioxidant protection during the critical early phase of UVB exposure.

### 2.6. Cytotoxicity of UVB, Vehicle, Propolis Extract, FFS, and Reference Markers on HaCaT Keratinocytes

**Figure 6:**
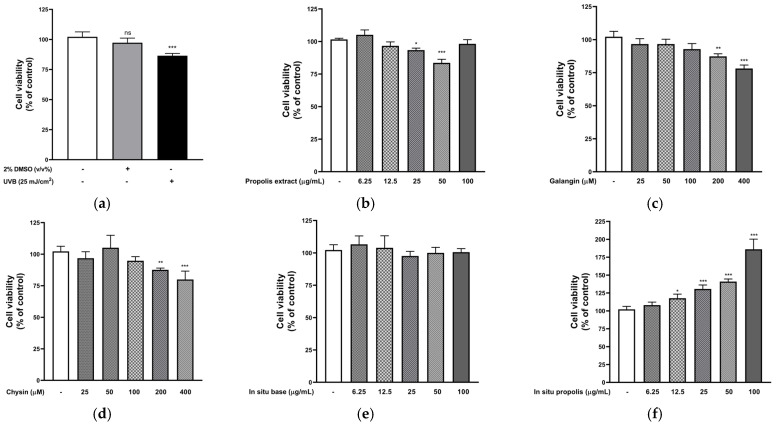
The cell viability of UVB and vehicle (**a**), propolis extract (**b**), galangin (**c**), chrysin (**d**), base FFS (BS) (**e**), and FFS-F7 (**f**) on HaCaT keratinocytes. Data represent triplicate experiments analyzed using one-way ANOVA (Dunnett’s post hoc test). The results represent the mean ± SD (* *p* < 0.05, ** *p* < 0.01, *** *p* < 0.001 vs. control group; ns: not significant).

UVB radiation (25 mJ/cm^2^) significantly reduced HaCaT cell viability by more than 20% (*p* < 0.001). In contrast, the vehicle control (2% dimethyl sulfoxide, DMSO), used as the solvent for the propolis extract, showed no significant effect on cell viability (Figure 6a). Treatment with propolis extract at 25 and 50 µg/mL significantly restrained cell proliferation (*p* < 0.05), whereas no significant effect was observed at the highest dose (100 µg/mL) (Figure 6b); the in situ base solution at concentrations of 6.25–100 µg/mL (Figure 6e), as well as galangin (Figure 6c) and chrysin (Figure 6d) at concentrations of 25–100 µM, did not significantly affect HaCaT cell viability when compared to the untreated group. These findings indicated that the vehicle, base formulation, and reference markers within the tested concentration ranges exhibited a nontoxic effect on HaCaT proliferation. Interestingly, FFS-F7 at concentrations of at least 12.5 µg/mL significantly enhanced HaCaT cell proliferation, indicating a potential stimulatory effect on HaCaT keratinocyte growth (Figure 6f).

### 2.7. Propolis Suppresses UVB-Induced DNA Damage Through Cell Cycle Arrest

**Figure 7:**
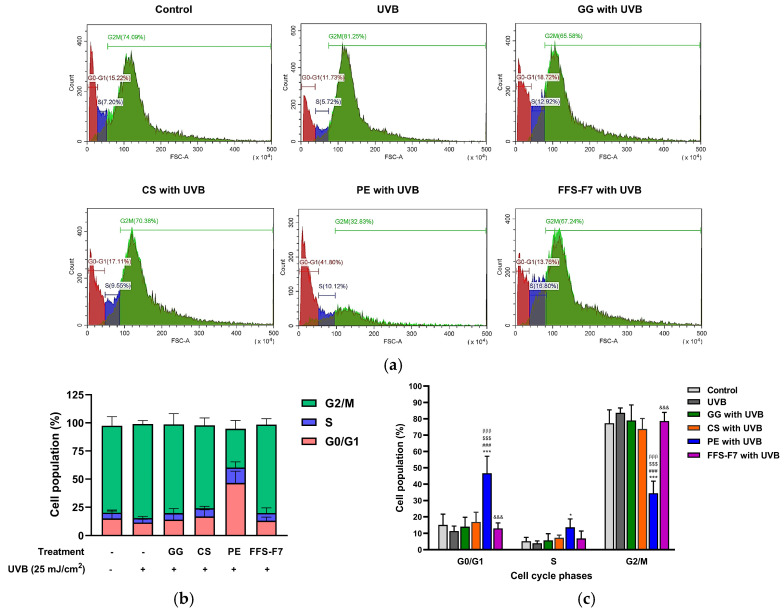
Propolis extract and FFS-F7 alleviate UVB-induced cell cycle distribution. (**a**) Representative histograms of the flow cytometry cell cycle analysis of different treatments. (**b**) Effect of various treatments on the cell cycle distribution of UVB-irradiated HaCaT cells. The percentages of cells in the G0/G1 (pink), S (blue), and G2/M (green) phases are shown as mean ± SD (n = 3). (**c**) Quantitative analysis of cell cycle distribution in UVB-irradiated HaCaT cells treated with galangin, chrysin, propolis extract, or FFS-F7 is represented by the percentage of cells in the G0/G1, S, and G2/M phases (mean ± SD, n = 3). Statistical significance was determined using two-way ANOVA and the Bonferroni multiple comparisons test. (* *p* < 0.05, *** *p* < 0.001 vs. control; ^###^ *p* < 0.001 vs. UVB; ^$$$^ *p* < 0.001 vs. galangin; ^βββ^ *p* < 0.001 vs. chrysin; ^&&&^ *p* < 0.001 vs. propolis extract). **Abbreviations:** GG: galangin; CS: chrysin; PE: propolis extract; FFS-F7: in situ propolis formulation.

To confirm whether the alterations in DNA damage–mediated cell death were caused by cell cycle arrest, HaCaT cells were treated with different concentrations of the test compounds for 24 h, followed by flow cytometry analysis of the cell cycle distribution. UVB irradiation at 25 mJ/cm^2^ decreased the proportion of cells in the G0/G1 phase (11.43% ± 2.89%) compared with control cells (15.02% ± 6.61%), suggesting either accelerated progression into subsequent phases of the cell cycle or induction of cell death. The S phase in UVB-irradiated cells was reduced (3.97% ± 1.38% of the cell population) compared to control cells (5.15% ± 2.41% of the cell population) (Table 4). Treatment with galangin, chrysin, propolis extract, and FFS-F7 increased the cell populations (%) in the G0/G1 and S phases compared to UVB-irradiated cell populations (Figure 7a,b), suggesting that these compounds and the formulation may protect against UVB-induced alterations in cell cycle progression at the G0/G1 and S phases. According to Figure 7c, which presents the cell cycle distribution as the percentage of cells in each phase following compound treatments, the propolis extract appears to exert a significantly protective effect by promoting cell cycle arrest at the G0/G1 phase (*** *p* < 0.001 vs. control; ^###^ *p* < 0.001 vs. UVB; ^$$$^ *p* < 0.001 vs. galangin; ^βββ^ *p* < 0.001 vs. chrysin). In parallel, the cell population in the G2/M phase after propolis extract treatment was significantly decreased compared to both the untreated group and the groups treated with other compounds, suggesting a halt in the mitotic division process (*** *p* < 0.001 vs. control; ^###^ *p* < 0.001 vs. UVB; ^$$$^ *p* < 0.001 vs. galangin; ^βββ^ *p* < 0.001 vs. chrysin). Conversely, as shown in Figure 7c, treatment with the FFS-F7 formulation resulted in a cell cycle distribution distinct from that observed with propolis extract. The proportion of cells in the G0/G1 phase was significantly reduced compared with propolis extract treatment, whereas the G2/M population was significantly increased (*p* < 0.001 vs. propolis extract). This redistribution suggests increased accumulation of cells in the G2/M phase together with a reduced proportion of cells in the G0/G1 phase following FFS-F7 treatment. Thus, FFS-F7 may provide complementary protective effects beyond those of propolis extract, highlighting the potential of formulation-based strategies to influence cellular responses to UVB-induced stress. Without UVB irradiation, differential effects between the propolis extract and FFS-F7 were evident in the dose-response analysis of cell viability (Figure 6b,f). Propolis extract reduced cell viability with increasing concentration, whereas FFS-F7 enhanced viability in a dose-dependent manner, highlighting distinct cellular responses to the two treatments under baseline conditions. These contrasting cell viability responses may contribute to the distinct cell cycle distributions observed with propolis extract under UVB exposure, resulting in a higher proportion of cells in the G0/G1 phase, whereas FFS-F7 treatment was associated with a higher proportion of cells in the G2/M phase compared with the propolis extract. These distinct outcomes suggest that the crude propolis extract and the formulated fraction FFS-F7 may act through fundamentally different mechanisms. Future studies should investigate the mechanistic basis of these differences, particularly the roles of compound composition.

### 2.8. Propolis-Loaded FFS Enhances Wound Healing in UVB-Induced HaCaT Cells

**Figure 8:**
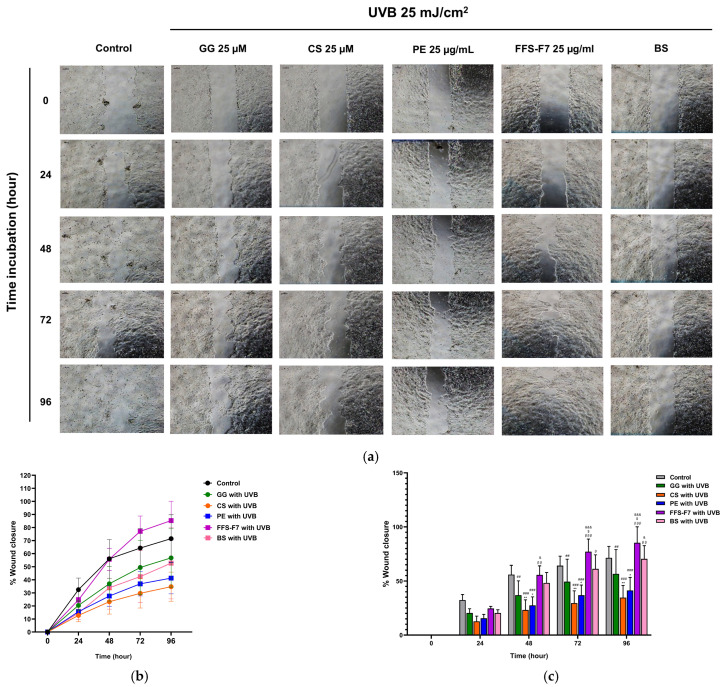
The wound healing potentials of propolis extract, FFS-F7, and reference markers were assessed by wound closure following UVB irradiation at different time points (0, 24, 48, 72, and 96 h). (**a**,**b**) The wound healing potentials of propolis extract (PE), FFS-F7, base in situ (BS), galangin (GG), and chrysin (CS). The base in situ (BS) represents the polymer formulation without propolis extract and was used as a formulation control. (**c**) Quantitative analysis of the number of migrating cells at different times. The control group represents PBS-treated cells without UVB irradiation. All data represent the mean ± SEM (n = 3). Statistical analysis was performed using two-way ANOVA followed by Bonferroni’s post hoc test (* *p* < 0.05, ** *p* < 0.01 vs. control; ^##^ *p* < 0.01, ^###^ *p* < 0.001 vs. UVB; ^$^ *p* < 0.05 vs. galangin; ^β^ *p* < 0.05, ^ββ^ *p* < 0.01, ^βββ^ *p* < 0.001 vs. chrysin; ^&^ *p* < 0.05, ^&&&^ *p* < 0.001 vs. propolis extract).

To isolate the vehicle contribution in FFS-F7, wound closure was additionally evaluated using the base FFS (base in situ) in UVB-irradiated cells. UVB irradiation at 25 mJ/cm^2^ delayed wound closure compared with the untreated control cells (Figure 8a), suggesting that UVB caused impairment of cellular function during the wound healing process across all time points (0, 24, 48, 72, and 96 h). Quantitative analysis of the wound gap (Figure 8b) demonstrated that FFS-F7 exhibited the highest wound closure progression rate compared with the untreated control cells and cells treated with the other compounds. Notably, FFS-F7 significantly enhanced the wound closure rate (%), indicating its efficacy in restoring UVB-induced impairment of wound healing relative to galangin, chrysin, and propolis extract (^$$$^ *p* < 0.001 vs. galangin; ^βββ^ *p* < 0.001 vs. chrysin; ^&&&^ *p* < 0.001 vs. propolis extract). In contrast, treatment with propolis extract alone did not significantly improve wound healing closure following UVB irradiation (Figure 8c).

## 3. Discussion

Propolis, a bee-derived product, has a long history of traditional and complementary use for mitigating skin inflammation and related conditions. Its pharmacological properties are considered to reflect the content of flavonoids and phenolic acids found in various types of propolis, particularly those produced by *Apis mellifera* bees. Flavonoids and phenolics are the primary bioactive compounds responsible for the antioxidant and anti-inflammatory effects, but their constituents and types in propolis vary based on several factors, such as biogeographic diversity, bee species, harvesting methods, seasonal changes, and extraction techniques. These variations likewise impact propolis quality and therapeutic potential in pharmaceutical applications [15,27].

In this study, *Apis mellifera* propolis was sourced from Chiang Rai and Phayao provinces in northern Thailand. HPLC analysis identified chrysin and galangin as the major flavonoid constituents, aligning with a previous report by Mukaide et al. (2021), who characterized and identified diverse prenylflavonoids using nuclear magnetic resonance imaging of *Apis mellifera* propolis from Chiang Mai, structurally related to chrysin and galangin [28]. In addition, caffeic acid phenethyl ester (CAPE) was detected in the present study and has been reported to possess antioxidant, anti-inflammatory, and anticancer activities (Figure 1b and Table 1). Galangin, chrysin, and caffeic acid phenethyl ester modulate key transcription factors, such as NF-κB and Nrf2, to inhibit the release of numerous pro-inflammatory cytokines and enhance antioxidant defenses in human keratinocytes exposed to UV radiation [29,30,31]. Supporting its biological effects, the propolis extract exhibited measurable total phenolic content (18.70 mg GAE/g DWE) and total flavonoid content (3.01 mg QE/g DWE), indicating the presence of bioactive compounds associated with their antioxidant and anti-inflammatory properties. However, the free radical scavenging by propolis extract indicated moderate activity (IC_50_ = 54.02 µg/mL), suggesting that antioxidant activity may be influenced by the composition and interactions of specific constituents rather than the total phenolic and flavonoid levels alone (Figure 2a and Table 1). This result agrees with earlier findings by Prior et al. (2005), who reported that total phenolic content was not necessarily correlated with a proportionally enhanced hydrophilic antioxidant capacity due to the variable efficacy of the individual phenolic constituents [32,33]. Similarly, nitric oxide (NO), a reactive nitrogen species involved in inflammation and oxidative stress, exhibited moderate scavenging activity in the propolis extract (IC_50_ = 81.72 µg/mL), reflecting a direct chemical interaction with NO radicals generated in the assay system. Importantly, further cell-based studies are warranted to determine whether these chemical scavenging effects translate into modulation of nitric oxide production and related inflammatory pathways in UVB-induced HaCaT keratinocytes (Figure 2c,d and Table 1) [33,34]. These results suggest that optimized film-forming systems may be required to improve the applicability of propolis extracts, particularly in addressing limitations associated with the relatively high concentrations needed for efficacy in traditional dosage forms.

All formulations exhibited a pH in the range of 5.5–7. This range closely approximates the normal skin pH, which is 4–7.4 depending on the anatomical site [35]. Furthermore, there are no reports of skin irritation occurring within this pH range. Consequently, the pH values of all formulations were within a safe, non-irritating range, making them suitable for topical application. Since n-butyl cyanoacrylate is a water-insoluble polymer that dissolves in organic solvents, it undergoes rapid polymerization and aggregation upon contact with water [36,37]. As it is mentioned that in situ forming systems are systems that can be easily transformed (ready to use), their limitation is stability. These results showed that adding rapid polymerization material, such as n-butyl cyanoacrylate, resulted in more sensitivity to the environment, even though the preparation showed it was well dissolved. Therefore, formulations F8–F14 were excluded from further formulation and film-forming evaluations.

The film character was directly related to the stability of FFS in the solution state, where the clear film was a result of stable FFS, while the translucent one was related to the turbid liquid of the n-butyl cyanoacrylate series. This difference is likely associated with variations in solvent composition and polymer interactions, which influence solvent evaporation rates and film formation kinetics; namely, the nuclei of n-butyl cyanoacrylate disrupted the Eudragit film formation [38]. Among the stable formulations, F7 exhibited the shortest drying time, significantly lower than that of F1 (Figure 5a). This behavior can be attributed to its higher ethanol content and lower dimethyl sulfoxide (DMSO) concentration, which enhances solvent volatility and accelerates film formation. In contrast, increased proportions of less volatile solvents, such as DMSO, tend to retard solvent evaporation and prolong drying time. The results were related to the other mechanism of in situ forming systems, solvent exchange. It was reported that a high concentration of the lower water-miscible solvent resulted in a slow in situ-forming process [39,40]. According to the drying time of F1–F7 preparations, it was found that the increased concentration of Eudragit^®^ RS 100 resulted in a significant decrease in the drying time of the film (Figure 4). However, the increased concentration of NMP in cosolvent resulted in a non-significant increase in the drying time. Because Eudragit RS^®^ 100 is the polymer that acts as a film-forming agent in the formulation through a crosslinking mechanism, having a high amount of Eudragit RS^®^ 100 in the formulation makes the film formation occur better. In addition, the high concentration of Eudragit^®^ RS 100 means there is less solvent in the formulation. This allows the solvent in the formulation to evaporate completely in a rapid manner, thus promoting faster film formation than the formulation with more solvent at the same time. Furthermore, increasing the concentration of Eudragit^®^ RS 100 significantly reduced the drying time of the film (Figure 4). However, the increased concentration of NMP in cosolvent resulted in a non-significant increase in the drying time. Because Eudragit RS^®^ 100 is the polymer that acts as a film-forming agent in the formulation through a crosslinking mechanism, having a high amount of Eudragit RS^®^ 100 in the formulation makes the film formation occur better. In addition, the high concentration of Eudragit^®^ RS 100 means there is less solvent in the formulation. This allows the solvent in the formulation to evaporate completely in a rapid manner, thus promoting faster film formation than the formulation with more solvent at the same time [41]. For mechanical properties evaluation, the increased %NMP in cosolvent and %Eudragit^®^ RS 100 resulted in a significantly higher penetration force of the film. Formulations F2, F5, and F6, which are formulations with the same solvent proportion and amount of Eudragit^®^ RS 100, had similar penetration force. While F1, F3, and F4 had negative penetration force, meaning the penetration force of the film on agar gel was lower than that of the single agar gel. Because these formulations contain only 2 types of solvents, which are Ethanol and DMSO, they might cause the obtained film to lack mechanical strength and gas and liquid permeation resistance from NMP. NMP is a synthetic compound in the pyrrolidone group that has the highest crosslinking ability compared to other solvents that can crosslink, such as DMSO [42]. Combined with DMSO, which has the ability to destroy hydrogen bonds, which are the bonds that form the gel. When spraying the formulation containing DMSO onto the agar gel, it decreases the strength of the agar gel. Therefore, the lack of NMP in the formulation is the factor that reduces the penetration force of the film on agar gel to be lower than the blank agar gel. It was found that formulation F7 has the highest penetration force at 17.358 g because it contains the highest NMP in cosolvent at 7% and also has the highest Eudragit^®^ RS 100 at 25%*w*/*w*. Eudragit^®^ RS 100 is a film-forming polymer through a crosslinking mechanism, so a high % Eudragit^®^ RS 100 will create dense crosslinking. This results in the highly flexible and strong film, which leads to the high penetration force. High WVTR indicated a high amount of water vapor can permeate through the film. The WVTR of normal skin is about 204 g/m^2^d. In the case of use, it is recommended that WVTR for wound dressing should have a higher value than normal skin. This is to allow water vapor exchange between air and skin and prevent the skin tissue from being too dry [43]. Hence, all formulations had higher WVTR than the normal skin value and were suitable. The increased % Eudragit^®^ RS 100 resulted in a significant decrease of WVTR. But the increased %NMP in cosolvent resulted in a non-significant decrease of WVTR (Figure 4c). As it was mentioned above, Eudragit^®^ RS 100 can form a film through a crosslinking mechanism. Therefore, having a high amount of Eudragit^®^ RS 100 in the formulation made the crosslinking mechanism occur more densely, leading to less water vapor permeating through the film. In addition, the structure of Eudragit^®^ RS 100, which has a carbonyl group, allows it to form a hydrogen bond with water vapor. This helps delay the permeation of water vapor, thus resulting in the decreased WVTR. Therefore, formulation F7, which is the formulation with the highest %Eudragit^®^ RS 100, had the lowest WVTR [44].

In addition to supporting previous studies, the present study provides a novel demonstration that in situ FFS, particularly sprays for topical applications, may represent a promising strategy for developing natural product-based pharmaceuticals that offer controlled delivery of bioactive compounds extracted from medicinal plants or polyherbal formulas [26,45]. Our optimal propolis-loaded FFS resulted in clear, homogeneous, and stable liquid dispersions with a pH range of 6.0–7.0, indicating its suitability for use as a topical medication (see Section 2.4). Regarding the FFS-F7 formulation, the use of ethanol facilitated rapid drying due to enhanced solvent evaporation (Figure 5a), whereas the use of dimethyl sulfoxide improved active compound solubility and film plasticity, ensuring the formation of a smooth and flexible propolis-loaded film [46]. Furthermore, formulating the propolis extract into an FFS enhanced its antioxidant efficacy compared to that of the propolis extract alone (Figure 5b,c). The use of Eudragit RS^®^ 100, a polymer used primarily for its film-forming capability, enabled effective compound encapsulation, thereby stabilizing and increasing the antioxidant potential. This encapsulation may preserve the structural integrity of phenolic and flavonoid compounds, maintaining their free radical scavenging abilities during storage and application [47]. The rapid antioxidant release of FSS-F7 (Figure 5d) also indicated that the Eudragit RS^®^ 100 polymer matrix provides immediate release. Since rapid in situ film formation is commonly associated with physical instability due to potential polymer precipitation during solvent evaporation, the short-term physical stability of the developed formulations was initially assessed. The prepared formulations were visually monitored for phase separation or precipitation within the first 24 h after preparation. Notably, the optimized FFS-F7 remained clear and homogeneous without visible precipitation or phase separation throughout the 24 h observation period, indicating acceptable short-term physical stability. Furthermore, the immediate action of antioxidant activity from the FFS-F7 matrix facilitates the rapid establishment of a protective barrier upon application. This characteristic is particularly advantageous for acute UVB exposure, where immediate neutralization of reactive oxygen species is critical to mitigate early-stage oxidative damage before the initiation of harmful biological cascades.

A further cellular mechanism of the study is the involvement of cell cycle regulation in propolis-mediated responses. The cell cycle, as a regulated sequence of cell growth including DNA replication and cell division, comprises two main phases: interphase and mitosis (M phase). Interphase is the stage during which the cell grows and prepares for division. Typically, mammalian cells divide within a 24 h cycle. The G0/G1 phase (responsible for metabolic activity and growth) takes approximately 9 h, the S phase (the DNA synthesis step) spans 10 h, the G2 phase lasts roughly 4.5 h, and the M phase occupies about 0.5 h. Our results for keratinocyte cytotoxicity using the MTT assay (Figure 7), which reflects cell viability through the activity of NAD(P)H-dependent oxidoreductase enzymes, demonstrated that UVB irradiation induced G2/M phase arrest in HaCaT keratinocytes (Figure 8a–c). This finding supports previous demonstrations that UVB-induced DNA damage activates cell cycle checkpoints, particularly at the G1/S and G2/M boundaries, as a cellular response to the propagation of DNA damage and cell apoptosis [48,49]. Our flow cytometry analysis also revealed that both the propolis extract and its FFS effectively neutralized UVB-induced cell cycle arrest in HaCaT keratinocytes (Figure 8a–c and Table 4). Surprisingly, the propolis extract induced a marked increase in the cell population in the G0/G1 phase, whereas FFS-F7 enhanced the S phase and reduced G2/M accumulation. This differential modulation of cell cycle distribution by the propolis extract and FFS-F7 indicated that the FFS may influence the cellular uptake or bioavailability of specific bioactive constituents. This leads to a comprehensive photoprotective effect that addresses multiple checkpoints in the cell cycle, thereby enhancing the overall resilience of keratinocytes against UVB-induced DNA replication stress. Moreover, the FFS-F7 effects were also significantly superior to those induced by galangin or chrysin, highlighting the critical role of the advanced film-forming delivery system in enhancing the diverse photoprotective mechanisms of propolis. Both the propolis extract and FFS-F7 protected normal keratinocytes against UVB-induced DNA damage by decreasing DNA replication stress and suppressing the cell cycle progression of DNA-damaged or mutagenic cells into mitosis—a process that leads to photocarcinogenesis [3]. Previous studies on the phenolic and flavonoid compounds in propolis extract have also demonstrated reductions in intracellular reactive oxygen species, the breakdown of DNA strands, and apoptosis in HaCaT keratinocytes following UV radiation exposure [50,51]. Nevertheless, the mechanisms of action by which propolis extract, FFS-F7, or the predominant bioactive markers (caffeic acid phenethyl ester, galangin, and chrysin) modulate cell cycle checkpoint activations and underlying pathways, such as G1/S checkpoint, p53/p21, ATR-Chk1/Chk2, Nrf2 signaling, and CDK-cyclin regulation, need further insightful investigations [52,53,54].

The wound healing efficacy of FFS-F7 was significantly enhanced (Figure 8), consistent with observations that UVB-induced cell cycle arrest in HaCaT keratinocytes substantially impairs wound healing by restricting cell proliferation and migration, essential processes for effective wound closure [50,55]. Interestingly, the enhanced wound closure observed in FFS-F7 suggests a potential synergistic interaction between the physical scaffold provided by the Eudragit^®^ RS100 matrix and the biochemical protection conferred by the propolis extract. While the film-forming polymers likely provide a favorable microenvironment for cell migration, the incorporation of propolis may further protect keratinocytes from UV-induced oxidative stress, thereby preserving the integrity of the regenerated tissue (Figure 8c). These observations indicated that Eudragit^®^ RS 100 and related film-forming polymers contribute to wound healing primarily through modulation of drug release and bioavailability of active compounds, as well as the formation of a protective barrier [56], rather than through direct biological activity. In parallel, propolis-derived constituents have been reported to modulate the expression of matrix metalloproteinases (MMP-1, -3, -7, and -9), which may partly contribute to the enhanced wound closure in UVB-irradiated HaCaT cells [50].

These findings warrant further investigation to validate the proposed mechanisms, elucidate the molecular pathways underlying the modulated antioxidant, anti-inflammatory, and skin homeostasis property-modulating activities, and assess their potential for clinical translation in human skin, particularly for photoprotection and the management of sensitive skin. Collectively, these observations highlight the potential therapeutic relevance of the formulation and provide mechanistic support for further evaluation in animal and clinical models, including long-term assessment of its photoprotective efficacy. Nevertheless, further in vivo studies are required to evaluate additional aspects such as immune interactions, skin barrier function, tolerability, and long-term photoprotective efficacy under physiologically relevant conditions.

## 4. Materials and Methods

### 4.1. Chemicals, Reagents, Equipment, and Tools

All reagents were of analytical grade. Standard compounds included L-ascorbic acid, caffeic acid phenethyl ester, p-coumaric acid, quercetin, chrysin, galangin, gallic acid, and 2,2-diphenyl-1-picrylhydrazyl (DPPH) (Sigma-Aldrich, St. Louis, MO, USA). The in situ film-forming reagents: n-butyl cyanoacrylate (Henkel, Shanghai, China), Eudragit^®^ RS 100 (Evonik Nutrition & Care GmbH, Essen, Germany), N-methyl-2-pyrrolidone (LOBA Chemie, Mumbai, India), dimethyl sulfoxide (Fisher Scientific UK, Loughborough, UK), absolute ethanol (QRëC, Auckland, New Zealand), di-sodium hydrogen phosphate anhydrous (Carlo erba reagent, Milan, Italy), and sodium hydroxide were purchased. A pH-meter (Inolab, Weilheim, Germany), rheometer (Brookfield, Middleboro, MA, USA), texture analyzer (Stable micro system, Surrey, UK), Vacuum Desiccator (JCT, Foshan, China), shaking incubator (N-BIOTEK, Bucheon-si, Republic of Korea), microplate reader Sp (BMG Labtech, Ortenberg, Germany), filter paper (Whatman™ No. 1, Schleicher&Schuell, Maidstone, UK), glass slide (SAIL, Shanghai, China), analytical balance (SCIENTIFIC, Bangkok, Thailand), autopipette (Nichiryo, Tokyo, Japan), and digital micrometer (SHAHE, Ningbo, China) were used.

### 4.2. Sample Collection and Quality Assessment

*Apis mellifera* propolis samples, sourced between June 2023 and October 2024, were purchased from industrial-scale agricultural beekeepers in Chiang Rai and Phayao provinces, northern Thailand. The samples were stored in silver polyethylene bags and preserved at temperatures ranging from −20 to 4 °C. Quality assurance tests of the raw material and extract were conducted in accordance with the Herbal Product Act B.E. 2562 (2019) (National Drug System Development Committee, 2019) [57], the Thai Herbal Pharmacopoeia (Department of Medical Sciences, 2000) [58], and the U.S. Pharmacopeia (USP) 61, including microbial enumeration tests. Organoleptic tests exhibited a solid, black, glossy, brittle, non-sticky substance with a strong characteristic odor. Microscopic analysis revealed numerous oil droplets without pollen (Figure S1). The overall quality profile, including limit tests and microbial and heavy metal contamination, is summarized in Table S1.

### 4.3. Propolis Extract Preparation

Propolis samples were stored in a −20 °C freezer for at least 24 h and ground into a fine powder. The powdered material was mixed with 70% ethanol (L PURE 95, Liquor Distillery Organization, Chachoengsao, Thailand) at a 1:10 (*w*/*v*) ratio and shaken at 120 rpm for 72 h at room temperature. After filtration through No. 2 Whatman filter paper (Cytiva, Marlborough, MA, USA), the extract was centrifuged at 5000× *g* for 5 min at 4 °C (Thermo Fisher Scientific, Waltham, MA, USA). The propolis extract was concentrated in a rotary evaporator (Buchi, Flawil, Switzerland) at 40–45 °C under 90–150 mbar, stored at −20 °C overnight, and vacuum-dried at 50 °C for 40 ± 5 h (Binder, Tuttlingen, Germany).

### 4.4. Determination of Total Phenolic and Flavonoid Contents

The total phenolic content (TPC) was determined by the Folin–Ciocalteu assay [59]. A stock solution of propolis extract (10 mg/mL in DMSO) was prepared and subsequently diluted to a working concentration of 50 μg/mL. Briefly, 20 μL of the sample was mixed with 45 μL of Folin–Ciocalteu reagent and 135 μL of 2.0% (*w*/*v*) sodium carbonate (Na_2_CO_3_) in a 96-well plate. The mixture was gently mixed using a microplate shaker and incubated for 60 min in the dark. Absorbance was measured at 735 nm using a UV spectrophotometer (BMG LABTECH, Ortenberg, Germany). TPC was calculated from a gallic acid calibration curve (6.25–100 μg/mL; R^2^ = 0.9992) (Figure S2) and expressed as milligrams of gallic acid equivalents per gram of dried propolis extract (mg GAE/g DWE).

The total flavonoid content (TFC) was determined using a modified aluminum chloride colorimetric assay [60]. Propolis extracts were diluted in 50% DMSO to final concentrations of 1 mg/mL. Briefly, 0.3 mL of 5% (*w*/*v*) sodium nitrite was added, followed by 5 min incubation at room temperature. Then, 0.15 mL of 10% (*w*/*v*) AlCl_3_ was added, and the mixture was incubated for 30 min at room temperature. Absorbance was measured at 510 nm using a UV-Vis spectrophotometer, with TFC quantified using a quercetin standard curve (0.1–1.6 mg/mL; R^2^ = 0.9999) (Figure S3) and reported as mg QE/g DWE.

### 4.5. Identification of Bioactive Markers in Propolis Extract Using Reversed-Phase High-Performance Liquid Chromatography

Potential biomarkers in the propolis extract were identified by reversed-phase high-performance liquid chromatography (HPLC) [61] using gallic acid, p-coumaric acid, caffeic acid phenethyl ester, quercetin, chrysin, and galangin as screening markers. The Agilent 1260 HPLC system (Agilent, Santa Clara, CA, USA) equipped with a Mightysil RP-18 GP column (4.6 × 150 mm, 3.0 µm particle size) (Kanto Chemical, Tokyo, Japan) using gradient elution and mobile phases of a 1.0% acetic acid-water solution (solvent A) and absolute methanol (solvent B) at a flow rate of 0.8 mL/min with gradient conditions (Table 5). Chromatographic peaks were detected at 280 nm.

### 4.6. Determination of Antioxidants by DPPH Radical Scavenging Activity

The free radical scavenging activity was determined for serially diluted propolis extract (6.25–100 μg/mL) using L-ascorbic acid and gallic acid as positive controls. Samples were mixed with DPPH (0.1 mM in EtOH) in a 96-well plate and measured at 520 nm using the spectrophotometer [62].

### 4.7. Determination of Anti-Inflammation by Nitric Oxide Scavenging Activity

The nitric oxide scavenging effectiveness was evaluated in the diluted samples using gallic acid as the positive control [63]. The propolis extract was mixed with 10 mM sodium nitroprusside (Merck & Co., Inc., Rahway, NJ, USA) and incubated for 150 min. Then, 1% sulfanilamide in 2% H_3_PO_4_ (Sigma-Aldrich, St. Louis, MO, USA) was mixed and incubated for 5 min before measuring at 540 nm using the spectrophotometer.

### 4.8. Propolis-Loaded FFS Formulation and Evaluation

#### 4.8.1. FFS Formulation and Preparation

The formulation was designed using a statistical approach based on mathematical modeling, specifically a full factorial design of experiment (DoE) together with preliminary data, polymer (Eudragit), and co-polymer (n-butyl cyanoacrylate) dissolving in various ratios of mixed solvent systems (ethanol, NMP, and DMSO). The variables were indicated (Table 6), and the concentration of components was calculated (Table 7).

The formulation process was initiated by dissolving the polymers within the specified solvent systems. Eudragit^®^ RS 100 was dissolved in ethanol, while n-butyl cyanoacrylate, where applicable, was dissolved in a mixture of N-methyl-2-pyrrolidone (NMP) and dimethyl sulfoxide (DMSO). The resulting mixtures were agitated in a shaking incubator to ensure complete polymer swelling. Subsequently, the mixture containing n-butyl cyanoacrylate was transferred into the Eudragit^®^ RS 100 solution. The combined mixture was further agitated in a shaking incubator until a clear and homogeneous solution was obtained. Propolis was then incorporated into the solution, and the final volume was adjusted to the specified level using ethanol as the solvent.

#### 4.8.2. Evaluation of FFS Solution: Apparent Stability (24 h Assessment), pH, and Viscosity

The formulation was visually evaluated for clarity, color, precipitation, and phase separation after being maintained at rest in a desiccator for 24 h. The pH was measured using a pH meter (Innolab, Weilheim, Germany). Viscosity was determined using a rheometer (Brookfield R/S, Middleborough, MA, USA) at ambient temperature. A shear rate of up to 1000 s^−1^ was applied for 3 min, with measurements recorded at 10 s intervals. Results were expressed in centipoise (cP) (n = 3).

#### 4.8.3. Film-Forming Behavior (Drying Time) and Post-Drying Stickiness Evaluation

Film surface characteristics, including texture, elasticity, smoothness, and transparency, were visually evaluated. The film-forming process was assessed in a Petri dish to examine structural integrity, homogeneity, and potential polymer precipitation. Transparency, smoothness, and stickiness were evaluated according to the method described by Frederiksen et al. [56].

Drying time was determined on porcine skin (by-product from a local slaughterhouse). The formulation was sprayed onto the skin and visually monitored until complete solvent evaporation. The film was then gently pressed with a cotton swab; the time at which no cotton fibers adhered to the surface was recorded as the drying time. The post-drying stickiness was evaluated concurrently. The absence of cotton fiber adhesion indicated a non-sticky film, following the method described by Saudagar et al. [64].

#### 4.8.4. Mechanical Properties Evaluation

The puncture strength of the film was measured using a texture analyzer (Stable Micro Systems, Surrey, UK) equipped with a 2 mm stainless steel cylindrical probe. The test was performed at a constant speed of 10 mm/s, and the maximum puncture force (g) was recorded (n = 3). The puncture strength of blank agar gel was measured and compared with that of agar gel coated with the film-forming spray.

#### 4.8.5. Water Vapor Transmission Rate (WVTR)

WVTR was determined as the mass of water vapor permeating through the film per unit area per day. The film-forming formulation was sprayed onto Whatman™ No. 1 filter paper and allowed to dry. The coated paper was cut into a circular sheet and placed over the orifice of a glass vial containing 20 mL of distilled water, then tightly secured with a rubber band. The initial mass was recorded before storing the assembly in a vacuum desiccator under controlled humidity at room temperature for 72 h. After 72 h, the final mass was recorded, and the WVTR was calculated utilizing Equation (1) [65].

(1) WVTR=(W/A)×(24/t)

where: WVTR is the water vapor transmission rate (g/m^2^d) and W is the weight of water lost (g). A is the surface area of the vial orifice (m^2^); t is the exposure time (h).

#### 4.8.6. Antioxidant Activity and Time-Dependent Release of Propolis-Loaded FFS

The free radical scavenging activity of the propolis-loaded FFS and the antioxidant components released over time from the highest-performing propolis-loaded film were evaluated using the DPPH assay [62]. For sample preparation, 80 µL of a 0.5% propolis-loaded FFS was diluted with 960 μL of 95% ethanol, followed by serial dilutions to achieve final concentrations (6.25–100 µg/mL). The propolis-loaded FFS was applied onto a glass slide (25.4 × 76.2 mm) and allowed to dry completely. The dried film was immersed in 50 mL of phosphate buffer solution (pH 5.8) and incubated at 37 ± 0.5 °C with continuous shaking at 50 rpm on an orbital shaker. Samples were collected at predetermined time intervals (0, 0.5, 1, 2, 4, 6, 8, and 12 h) to determine the antioxidant potency of the released components.

### 4.9. Cell Culture and Treatments

The immortalized human keratinocyte cell line (HaCaT) was obtained from Elabscience^®^ (catalog no. EP-CL-0090, Elabscience Biotechnology Inc., Houston, TX, USA). Cells (passages 25–27) were cultured in T75 flasks in Dulbecco’s Modified Eagle’s Medium (DMEM)/F12 supplemented with 10% fetal bovine serum and 1% penicillin (Biowest, Riverside, MO, USA) 100 μg at 4 °C in a 5% CO_2_ incubator until reaching at least 80% confluency. Propolis extract, propolis-loaded FFS, and reference compounds were dissolved in 2% dimethyl sulfoxide and 70% ethanol, filtered through a 0.22 μm PVDF filter, and serially diluted with serum-free DMEM to final concentrations (3.25–100 μg/mL). Additionally, the active propolis compounds (chrysin and galangin) were tested at concentrations between 25 and 400 μM to validate their biological effects.

For cell-based experiments, the propolis-loaded FFS (solution state) was dispersed in serum-free DMEM/F12 and sonicated to ensure homogeneity. The fine particles were utilized as a representative model of the film matrix within a localized area. Due to the significantly increased surface area of the micro-particulate state, a higher proportion of component release was achieved compared to conventional release profiles. Subsequently, the resulting suspension, in which all components were expected to be fully released at their limits, was filtered and subjected to further analytical testing. The dispersion was centrifuged at 2000 rpm for 5 min to remove insoluble materials, and the supernatant was subsequently filtered through a 0.22 μm PVDF membrane to obtain a clarified solution suitable for cell treatment. These maximized the reaching out of all components from the formulation to be evaluated under aqueous conditions required for cell-based assays, reflecting its rapid-release antioxidant activity. This approach does not replicate film formation on the skin surface but enables evaluation of formulation-derived constituents while preserving the relevance of the FFS in terms of its release characteristics. Cells were subsequently incubated under HaCaT cell culture conditions, and the effects of the released compounds were assessed in wound healing and cell cycle assays.

HaCaT cells were exposed to 25 s of UVB, corresponding to the dose of 25 mJ/cm^2^. using a Philips TL20W/01RS lamp (Philips, HamburgM Germany, 280–315 nm, stimulating natural UVB light), and then immediately treated with the relevant samples and standards for 24 h. The energy output of UVB (290–320 nm) was measured with a UVB photometer (IL1350 photometer; International Light, Newburyport, MA, USA).

### 4.10. Cell Viability Assay

Cell viability of all test compounds was assessed using the 3-(4,5-dimethylthiazol-2-yl)-2,5-diphenyltetrazolium bromide (MTT) assay. Cells (5000 cells/well) were seeded into a 96-well plate until 80% confluent, exposed to UVB, and treated with varying concentrations of test compounds. After 24 h, MTT reagent (0.5 mg/mL in PBS) was added to each well and incubated at 37 °C for 4 h. Formazan crystals were solubilized with DMSO, and absorbance was measured at 570 nm using a UV-spectrophotometer. Cell viability was calculated as a percentage relative to untreated cells (n = 3) [66].

(2) $$\mathrm{Cell}\;\mathrm{viability}\;\left(\%\right)=\left[\frac{\mathrm{Abs}\;\mathrm{treated}\;\mathrm{cell}-\mathrm{Abs}\;\mathrm{culture}\;\mathrm{medium}}{\mathrm{Abs}\;\mathrm{untreated}\;\mathrm{cell}\mathrm{Abs}\;\mathrm{culture}\;\mathrm{medium}\;}\right]\;\times \;100$$

### 4.11. Cell Cycle Analysis Using Flow Cytometry

At 24 h post-UVB radiation, treated cells were harvested with 0.25% Trypsin-EDTA, fixed in 70% ethanol for 1 h at room temperature, washed twice with phosphate-buffered saline (Corning Life Sciences, Tewksbury, MA, USA), and then incubated with 1.5 mg/L RNAse A (Cell Signaling Technology, Inc., Danvers, MA, USA) for 1 h at 37 °C. After staining with 5 µL of propidium iodide (Cell Signaling Technology, Inc., Danvers, MA, USA) for 20 min in the dark on ice, the cell cycle phases were immediately assessed and analyzed using a CytoFLEX Flow Cytometer (Beckman Coulter, Inc., Brea, CA, USA). Analysis of cell cycle distribution and percentage of cells in G0–G1, S, and G2/M phases of the cell cycle was determined at a rate of 1200 events/s with excitation by a 488 nm laser beam. Untreated cells without UV exposure served as negative controls. The histograms were analyzed using CytoFLEX software version 2.5 (Beckman Coulter, Inc., Brea, CA, USA) to determine the percentage of cells in each phase of the cell cycle (n = 3). PI binds stoichiometrically to DNA, producing fluorescence intensity proportional to DNA content. In the resulting histogram, % cells population in G0/G1 appears as the first peak with diploid DNA, S-phase cells form a broad distribution between diploid DNA and tetraploid DNA due to ongoing replication, and G2/M cells appear as the second peak with tetraploid DNA [67,68].

### 4.12. Wound-Healing Assay

The wound-healing potential of the in situ propolis-loaded film-forming solution was evaluated using a scratch assay. HaCaT cells (150,000 cells/well) were seeded into 24-well plates and cultured for 72 h until they reached 100% confluency. Cells were exposed to UVB at 25 mJ/cm^2^, and then a uniform linear wound was created in the monolayer using a sterile 200 µL pipette tip. To remove detached cells and debris, the monolayer was washed twice with phosphate-buffered saline. The cells were then treated with samples or reference compounds. Wound closure was monitored and imaged at 0, 24, 48, 72, and 96 h post-treatment using an inverted light microscope (Olympus FV3000, Tokyo, Japan). The extent of wound healing was quantified by measuring the wound area (µm^2^) at each time point using ImageJ software (version 1.54p, National Institutes of Health, USA) with the FIJI function and expressed as a percentage of wound confluence (n = 3) [69,70].

(3) $$\mathrm{Wound}\;\mathrm{confluence}\;\left(\%\right)=\left[\frac{A-B}{A}\right]\times \;100$$

Calculation of wound confluence. A: the width of the initial scratch wound (0 h), B: the width of the scratch wound at time 24, 48, 72, or 96 h.

### 4.13. Statistical Analysis

All experiments were performed in triplicate (n = 3), and data are presented as mean ± standard deviations (SD) or standard error of the mean (SEM). Statistical analyses were conducted using GraphPad Prism version 8 for Windows (GraphPad Software Inc., San Diego, CA, USA). Comparisons among multiple groups were performed using one-way analysis of variance (ANOVA), followed by Bonferroni or Dunnett’s multiple comparison test, as indicated in the figure legends. For time-course and factorial analyses, two-way ANOVA with Bonferroni post hoc testing was applied. A *p*-value < 0.05 was considered statistically significant.

## 5. Conclusions

This study highlights the role of FFS to enhance the therapeutic potential of Thai *Apis mellifera* propolis. HPLC profiling revealed that galangin, chrysin, and caffeic acid phenethyl ester are the major bioactive constituents of the extract. The propolis-loaded FFS formulated with Eudragit^®^ RS 100 effectively attenuated UVB-induced skin damage by reducing cytotoxicity, facilitating cell cycle recovery, and enhancing wound closure in HaCaT cells. Moreover, the FFS formulation improved wound healing in HaCaT cells following UVB exposure. These findings indicate that the formulation could serve as a promising supportive topical strategy for mitigating cellular stress and supporting skin regeneration while addressing limitations of traditional dosage forms, such as poor skin residence time and undesirable sensory properties.

## Figures and Tables

**Table 1:** The percentage yields, total phenolic and flavonoid contents, and IC_50_ values for free radical scavenging and nitric oxide inhibitory activities of propolis extract. Data represent mean ± SD of triplicate experiments.

Name	% Yields (*w*/*w*)	Total Phenolic Content (GAE mg/g DWE)	Total Flavonoid Content (QE mg/g DWE)	DPPH Assay IC_50_ (µg/mL)	NO Assay IC_50_ (µg/mL)
L-ascorbic acid	-	-	-	9.75	-
Gallic acid	-	-	-	8.53	5.17
Caffeic acid phenethyl ester	-	-	-	6.61	20.72
Chrysin	-	-	-	>100	>100
Galangin	-	-	-	>100	>100
Propolis extract	51.19	18.70 ± 0.65	3.01 ± 0.05	54.02	81.72

(*w*/*w*) = gram unit. GAE: Gallic acid equivalent; QE: Quercetin; DWE: Dry weight extract.

**Table 2:** Identification of target phenolic and flavonoid markers identified in Thai propolis extract. Data represent mean ± SEM of triplicate experiments.

Compound Name	Amount (µg/mL)	%RSD	% *w*/*w*	Calibration Curve	R^2^
Phenolic markers					
Gallic acid	ND		-	-	-
p-coumaric acid	ND	-	-	-	-
Caffeic acid phenethyl ester	1.34 ± 0.01	0.81	1.34	y = 35627x + 18161	0.9997
Flavonoid markers					
Quercetin	ND	-	-	-	-
Chrysin	43.72 ± 0.06	0.15	43.72	y = 69542x + 18471	0.9996
Galangin	40.39 ± 0.08	0.19	40.39	y = 45117x + 423	0.9997

Limit of qualification (LOQ) and linearity were 0.05 µg/mL and 0.05–200 µg/mL, respectively. % *w*/*w*: mg/g dry extract; %RSD: relative standard deviation; ND: not detected.

**Table 3:** Physical properties, film-forming characteristics, pH values, and drying time of seven optimized propolis-loaded film formulations.

Formulation Code	Physical Properties	Film-Forming Characteristics		pH	Drying Time (min) (Mean ± SD)
		Physical Appearance	Film-Forming Appearance		
F1	Clear Homogeneous Absence of sediment and phase separation	Clear Homogeneous Smooth Flexible, conforming to the surface	Transparency Homogeneity Smooth texture Non-sticky property	6.0	4.98 ± 0.45
F2				7.0	3.72 ± 0.41
F3				6.0	3.24 ± 0.04
F4				6.0	5.22 ± 0.17
F5				6.0	3.41 ± 0.12
F6				6.0	3.63 ± 0.39
F7				6.0	3.08 ± 0.07
F8	Tubid liquid	Turbid film	Translucent Smooth texture Non-sticky property	5.5	36.22 ± 2.03
F9				5.5	15.44 ± 0.74
F10				5.5	6.82 ± 0.33
F11				5.5	5.95 ± 0.20
F12				5.5	16.08 ± 0.91
F13				5.5	39.28 ± 1.99
F14				5.5	15.23 ± 0.56

**Table 4:** Cell cycle distribution (G0/G1 phase, S phase, and G2/M phase). All data are presented as the mean ± SD of triplicate experiments.

Name	G0/G1	S	G2/M
Control*	15.02 ± 6.61	5.15 ± 2.41	77.31 ± 8.13
UVB	11.43 ± 2.89	3.97 ± 1.38	83.63 ± 2.99
GG with UVB	13.98 ± 5.70	5.74 ± 4.03	79.00 ± 9.45
CS with UVB	16.84 ± 5.92	7.29 ± 1.65	73.77 ± 6.41
PE with UVB	46.65 ± 10.40	13.63 ± 5.05	34.48 ± 7.35
FFS-F7 with UVB	13.04 ± 3.19	6.84 ± 4.54	78.60 ± 5.27

Control* = Untreated cells (negative control group). GG: galangin 100 µM; CS: chrysin 100 µM; PE: propolis extract 100 µg/mL; FFS-F7: in situ propolis formulation 7 100 µg/mL; UVB: Ultraviolet B radiation 25 mJ/cm^2^.

**Table 5:** HPLC gradient conditions for targeted reference markers in propolis analysis.

Time (min)	Percentage of Solvent		Flow Rate (mL/min)
	Solvent A	Solvent B	
0.00	0	100	0.8
0.01	80	20	0.8
8.00	70	30	0.8
12.00	50	50	0.8
20.00	50	50	0.8
25.00	40	60	0.8
35.00	25	75	0.8
45.00	0	100	0.8

**Table 6:** Experimental design (DoE): independent variables, levels, and response constraints for film-forming systems with and without n-butyl cyanoacrylate.

Condition	Variables	Level		
		[−1]	[0]	[+1]
Without n-butyl cyanoacrylate preparations	Independent Variables			
	Eudragit^®^ RS 100 (%*w*/*w*)	15.00	20.00	25.00
	%NMP in cosolvent mixtures *	0.00	3.50	7.00
	Dependent Variables			
	Penetration force (g)	Maximize		
	Drying time (min)	Minimize		
	Water Vapor Transmission Rate (g/m^2^d)	In range		
With n-butyl cyanoacrylate preparations	Independent Variables			
	Eudragit^®^ RS 100 (%*w*/*w*)	14.95	19.95	24.95
	%NMP in cosolvent mixtures *	0.00	3.50	7.00
	Dependent Variables			
	Penetration force (g)	Maximize		
	Drying time (min)	Minimize		
	Water Vapor Transmission Rate (g/m^2^d)	In range		

* NMP and DMSO cosolvent mixtures.

**Table 7:** Formulation composition of propolis-loaded FFS with and without n-butyl cyanoacrylate.

No.	Propolis (%*w*/*w*)	Eudragit^®^ RS 100 (%*w*/*w*)	n-Butyl Cyanoacrylate (%*w*/*w*)	Ethanol (%*w*/*w*)	NMP (%*w*/*w*)	DMSO (%*w*/*w*)	Total (%*w*/*w*)
F1	0.50	15.00	0.00	60.00	0.00	24.50	100.00
F2	0.50	20.00	0.00	60.00	0.68	18.82	100.00
F3	0.50	25.00	0.00	60.00	0.00	14.50	100.00
F4	0.50	15.00	0.00	60.00	1.72	22.79	100.00
F5	0.50	20.00	0.00	60.00	0.68	18.82	100.00
F6	0.50	20.00	0.00	60.00	0.68	18.82	100.00
F7	0.50	25.00	0.00	60.00	1.02	13.49	100.00
F8	0.50	24.95	0.05	60.00	0.00	14.50	100.00
F9	0.50	19.95	0.05	60.00	0.68	18.82	100.00
F10	0.50	14.95	0.05	60.00	0.00	24.50	100.00
F11	0.50	14.95	0.05	60.00	1.72	22.79	100.00
F12	0.50	19.95	0.05	60.00	0.68	18.82	100.00
F13	0.50	24.95	0.05	60.00	1.02	13.49	100.00
F14	0.50	19.95	0.05	60.00	0.68	18.82	100.00

## Data Availability

The original contributions presented in this study are included in the article/Supplementary Material. Further inquiries can be directed to the corresponding author.

## Supplementary Materials

The following supporting information can be downloaded at: https://www.mdpi.com/article/10.3390/ph19050680/s1, Figure S1: The macroscopic and microscopic characteristics of crude propolis; Table S1: The quality assurance profiles of propolis; Figure S2: Standard calibration curve of gallic acid for total phenolic content quantification; Figure S3: Standard calibration curve of quercetin for total flavonoid content quantification.
