# Supplementary material for: In Situ Thai Apis mellifera Propolis Film as Potential Protective Phytopharmaceuticals Against UVB-Induced HaCaT Keratinocyte Damage
Source: Pharmaceuticals (Basel). 2026 Apr 27;19(5):680. doi: 10.3390/ph19050680 (PMC13209595; doi:10.3390/ph19050680)

Supplementary material

Supplementary Figure S1. The macroscopic (A) and microscopic (B) characteristics of crude propolis

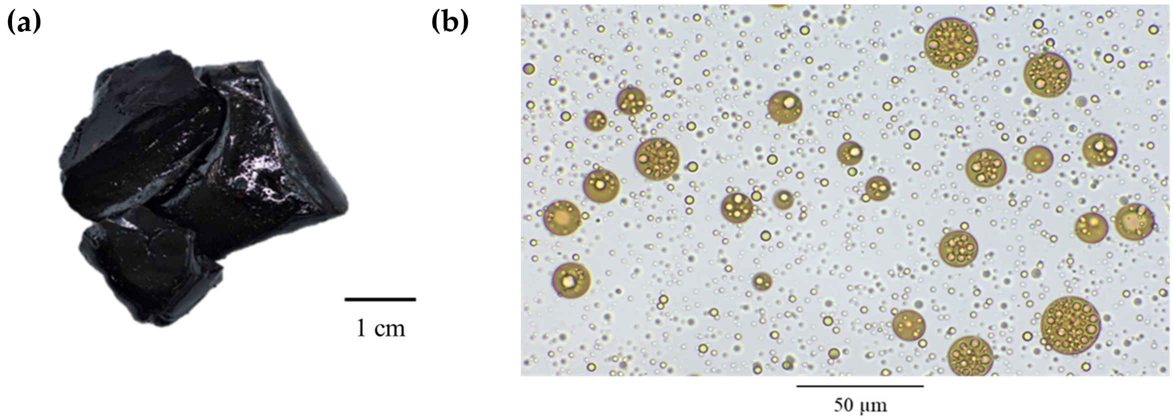

Supplementary Table S1. The quality assurance profiles of propolis.

Specification	Result	Guidelines and Standardization
**Limit Tests (Content%)**		
Moisture content	1.13 ± 0.15	maximum 6
Foreign matter	ND	less than 2%
Total ash content	0.17 ± 0.02	maximum 10
Wax content	0.18 ± 0.06	maximum 30
Resin content	98.34 ± 0.09	maximum 50
**Microbial contamination**		**(National Drug System Development Committee, 2019; US Pharmacopeia (USP), 2024)**
Total aerobic microbial count (cfu/g)	Pass	less than $2x10^2$
Total yeast and mold count (cfu/g)	Pass	less than $2x10^2$
Staphylococcus aureus	ND	no detected in 1 gram
Pseudomonas aeruginosa	ND	no detected in 1 gram
Clostridium spp.	ND	no detected in 1 gram
**Heavy metal contamination**		**(Department of Medical Sciences, 2000)**
Lead (Pb)	ND	no more than 10 ppm
Cadmium (Cd)	ND	no more than 0.3 ppm
Arsenic (As)	ND	no more than 4 ppm
Mercury (Hg)	ND	no more than 0.5 ppm

The levels of heavy metal contaminants in propolis extract were analyzed using Inductively Coupled Plasma Optical Emission Spectrometry (ICP-OES), and all data are presented as mean ± SD of triplicate experiments.

Abbreviations ND: not detected

Supplementary Figure S2. Standard calibration curve of gallic acid for total phenolic content (TPC) quantification by the Folin–Ciocalteu method. Gallic acid concentrations of 6.25, 12.5, 25, 50, and 100 µg/mL were applied. Linear regression: y = 0.0056x + 0.0344 (R^2 = 0.9992).

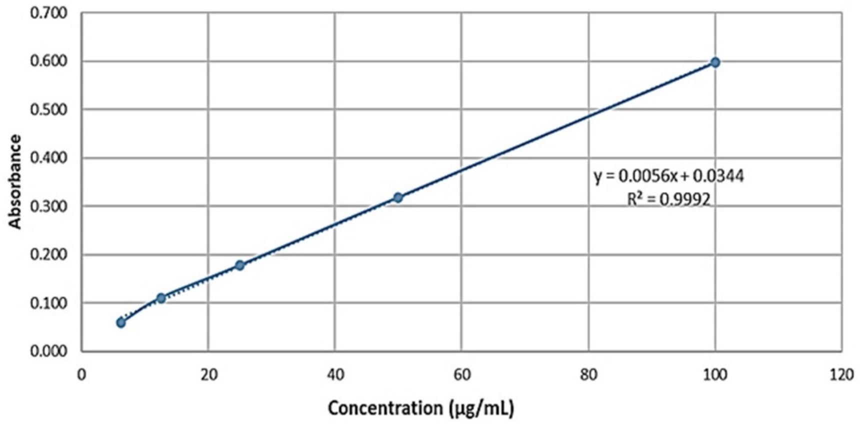

Supplementary Figure S3. Standard calibration curve of quercetin for total flavonoid content (TFC) quantification by the aluminum chloride colorimetric method. Quercetin concentrations of 0.1, 0.2, 0.4, 0.6, 0.8 and 1.6 mg/mL were used. Linear regression: y = 0.2824x + 0.0186 (R^2 = 0.9999).

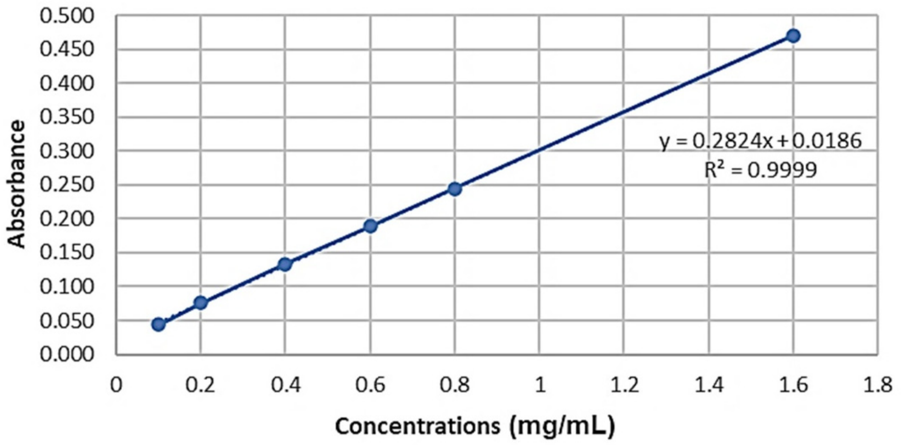

Supplement: Supplementary file 1 [file pharmaceuticals-19-00680-s001.zip › pharmaceuticals-4188490-supplementary.pdf]
